# Development and Validation of High-Throughput Bioanalytical Liquid Chromatography-Tandem Mass Spectrometry (LC-MS/MS) Method for the Quantification of Newly Synthesized Antitumor Carbonic Anhydrase Inhibitors in Human Plasma

**DOI:** 10.3390/molecules25235753

**Published:** 2020-12-06

**Authors:** Ahmed M. Abdel-Megied, Wagdy M. Eldehna, Mohamed A. Abdelrahman, Fawzy A. Elbarbry

**Affiliations:** 1 Pharmaceutical Analytical Chemistry Department, Faculty of Pharmacy Kafrelsheikh University, Kafrelsheikh City 33516, Egypt; dr_ahmed80@pacificu.edu; 2 School of Pharmacy, Pacific University Oregon, Hillsboro, OR 97123, USA; 3 Department of Pharmaceutical Chemistry, Faculty of Pharmacy, Kafrelsheikh University, Kafrelsheikh City 33516, Egypt; wagdy2000@gmail.com; 4 Department of Pharmaceutical Chemistry, Faculty of Pharmacy, Egyptian Russian University, Badr City, Cairo 11829, Egypt; mohamed.ashraf.eru@gmail.com

**Keywords:** LC-MS/MS, tandem mass, carbonic anhydrase inhibitors, antitumor, human plasma, bioanalytical validation

## Abstract

In the present study, a sensitive and fully validated bioanalytical high-performance liquid chromatography-tandem mass spectrometry (LC-MS/MS) method has been developed for the quantitative determination of three newly synthesized carbonic anhydrases inhibitors (CAIs) with potential antitumor activity in human plasma. The analytes and the internal standard (IS) were extracted using 1.5 mL acetonitrile from only 450 µL aliquots of human plasma to achieve the desired protein precipitation. Chromatographic separations were achieved on Phenomenex Kinetex^®^ C_18_ column (100 × 4.6 mm, 2.6 µm) using a binary gradient elution mode with a run time of less than 6 min. The mobile phase consisted of solvent (A): 0.1% formic acid in 50% methanol and solvent B: 0.1% formic acid in acetonitrile (30:70, *v*/*v*), pumped at a flow rate of 0.8 mL/min. Detection was employed using triple quadrupole tandem mass spectrometer (API 3500) equipped with an electrospray ionization (ESI) source in the positive ion mode. Multiple reaction monitoring (MRM) mode was selected for quantitation through monitoring the precursor-to-parent ion transition at *m*/*z* 291.9 → 173.0, *m*/*z* 396.9 → 225.1, *m*/*z* 388.9 → 217.0, and *m*/*z* 146.9 → 91.0 for AW-9a, WES-1, WES-2, and Coumarin (IS), respectively. Linearity was computed using the weighted least-squares linear regression method (1/*x*^2^) over a concentration range of 1–1000, 2.5–800, and 5–500 ng/mL for AW-9a, WES-1, and WES-2; respectively. The bioanalytical LC-MS/MS method was fully validated as per U.S. Food and Drug Administration (FDA) guidelines with all respect to linearity, accuracy, precision, carry-over, selectivity, dilution integrity, and stability. The proposed LC-MS/MS method was applied successfully for the determination of all investigated drugs in spiked human plasma with no significant matrix effect, which is a crucial cornerstone in further therapeutic drug monitoring of newly developed therapeutic agents.

## 1. Introduction

Carbonic anhydrases (CAs) are family of metallo-enzymes ubiquitously expressed in prokaryotes and eukaryotes. They play a crucial role in pH regulation through reversible rehydration of CO_2_ [1]. Five families have been discovered within carbonic anhydrases; from these five families, only α-CAs have been characterized to be predominantly present in the animal kingdom [2,3]. Regarding the α-class, sixteen diverse CA isoforms have been discovered so far with large variation in their subcellular localization and tissue distribution is present in the mitochondrial matrix [4,5]. In particular, the human (*h*) isoform CA IX is ectopically expressed in hypoxic tumors, thus acting as a key player in cancer cells’ survival, proliferation, and metastasis [6], and its inhibition has been suggested as a promising strategy for the treatment of human malignancies [7,8].

**Figure 1:**
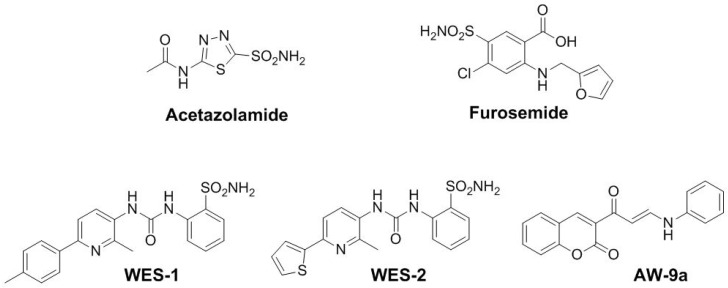
Chemical structures of the clinically approved (acetazolamide and furosemide) and newly synthesized (**WES-1**, **WES-2**, and **AW-9a**) carbonic anhydrase inhibitors.

There are several types of carbonic anhydrases inhibitors (CAIs) that exert their inhibitory action through different mechanisms. The primarily sulfonamide-based compounds [9,10] represent the classical CAIs that are widely used as zinc-binding groups (ZBGs) and include long-lasting clinically approved CAI-based molecules such as acetazolamide and furosemide (Figure 1) [11,12]. On the contrary, non-classical CAIs are not based on ZBGs such as phenols, polyamines, carboxylic acids, and coumarins. The latter group and its isosteres can inhibit CA through occluding the CA active site entrance and preventing catalysis [13,14,15]. Recently, compounds WES-1 and WES-2 (Figure 1) have been designed and synthesized as CA IX inhibitors. Both compounds displayed promising in vitro anti-proliferative activity. In addition, they possessed efficient in vitro anticancer activity towards colon cancer with apoptosis induction activity (unpublished results). Moreover, coumarin-based compound AW-9a (Figure 1) has been reported as a potent and selective tumor-associated CA isoform IX inhibitor endowed with promising activity against breast cancer (unpublished results).

To the best of the authors’ knowledge, the literature review revealed a limited number of liquid chromatography-tandem mass spectrometry (LC-MS/MS) research articles illustrating the determination of CAIs in human plasma [16,17,18,19,20], but no reported methods for the new CAIs under investigation. Herein, the present work is extremely essential to identify and quantify those newly synthesized antitumor drugs to develop a reliable validated LC-MS/MS bioanalytical method for simultaneous quantitation of all investigated drugs in pre-clinical and phase I clinical studies and further utilized for therapeutic drug monitoring (TDM) in a patient population.

## 2. Results and Discussion

### 2.1. Method Development and Optimization

**Figure 2:**
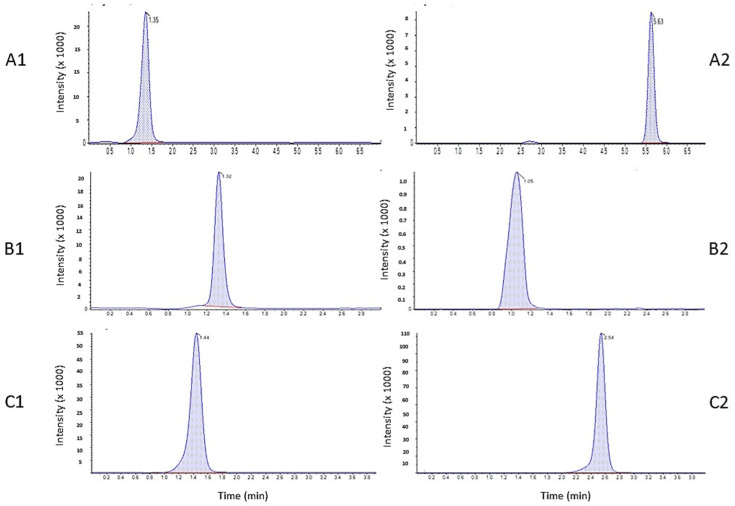
Representative mass chromatograms of blank human plasma spiked with 250 ng/mL IS (**A1**, **B1**, and **C1**) and 100 ng/ mL AW-9a (**A2**), 250 ng/mL WES-1 (**B2**), and 120 ng/mL WES-2 (**C2**).

The newly developed method should exhibit a high degree of sensitivity owing to the expected low target plasma concentration levels of the investigated drugs. Initially, several trials using different columns and mobile phases with different compositions and ratios were tried. A number of mobile phases consisted of mixtures of different ratios of organic modifier such as methanol, acetonitrile (30–90%), and acids such as glacial acetic acid (0.1–0.2%) and formic acid (0.05–0.2%), which were investigated to achieve improved resolution and lower retention times and diminish any matrix effect. Trials were performed via isocratic elution using 0.1% aqueous formic acid and ammonium formate at pH 3.0 with different ratios of acetonitrile and methanol. However, the results were unsatisfactory because of the apparent distortion and tailing of the tested analytes peaks due to high matrix interference. Optimum conditions were achieved with gradient elution consisting of solvent A: 0.1% formic acid in 50% water and 50% methanol and solvent B: 0.1% formic acid in acetonitrile (30:70, *v/v*), pumped at a flow rate of 0.8 mL/min according to the following flow program: 0.0–0.5 min (20% B), 0.5–1.5 min (15% B), 1.5–2.5 min (5% B), 2.5–3.5 min (30% B), 3.5–5.5 min (50% B), and 5.5–7.0 min (60% B), with optimum run time of less than 6.0 min at a flow rate of 0.8 mL/min using Kinetex C18 column (100 × 4.6 mm, 2.6 µm; Phenomenex, CA, USA) maintained at room temperature. Representative chromatograms of AW-9a, WES-1, and WES-2, respectively, are shown in Figure 2.

**Figure 3:**
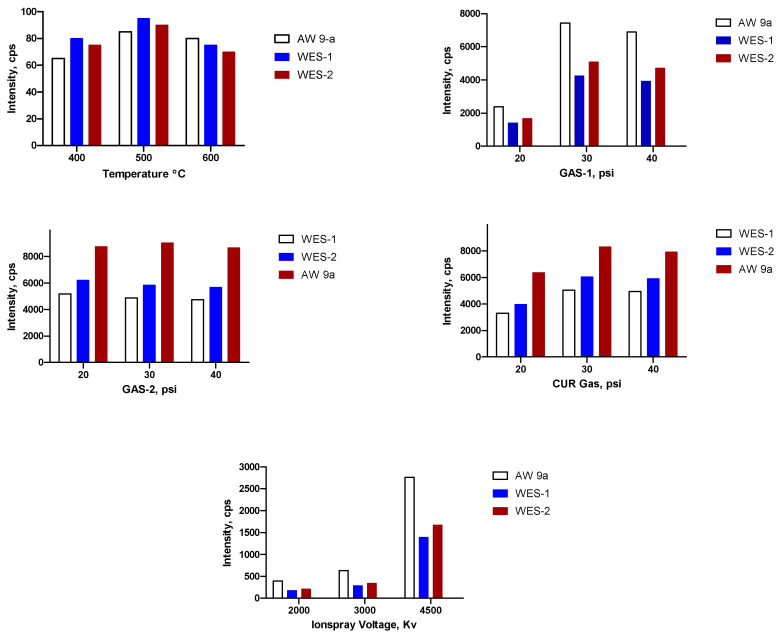
Optimization of the tandem mass spectrometry (MS/MS) parameters of the proposed method based on FIA MS/MS^ALL^ compound optimization tool in Analyst Hotfixes^TM^ software version 1.6.3.

It was challenging to assess the best mass spectrometric conditions for the newly synthesized drugs, namely, AW-9a, WES-1, and WES-2. Trials were carried out in both positive and negative ionization mode. It was found that the positive ionization mode provided a better response over the negative ionization mode, under different MS parameters. Therefore, it was selected in optimization parameters for monitoring the precursor as well as the product ions after direct infusion using the ABSciex infusion pump at a concentration of 500 ng/mL. The MRM transitions (*m/z*) were used in positive ionization mode to clear any potential interference signals and improve the sensitivity of the procedure. The optimized parameters included the following: collision energy (CE), declustering potential (DP), and collision cell exit potential (CXP) at a constant entrance potential at 10 V. CE is considered to be the most critical parameter for having reasonable daughter fragment ions responses. However, ramping up CE energy resulted in a rise in the intensity of the particular fragment ion till optimal values, during which a marked decline in the intensity would be observed, which could be detected owing to excessive unnecessary fragmentation for the targeted product ion, which is not recommended in such an MS/MS analysis. Furthermore, other MS/MS parameters were optimized for the greatest signal such as ionspray voltage, GS1, GS2, collision gas (CAD), and temperature of the ion source, and the results of the optimization studies are summarized in Figure 3.

**Figure 4:**
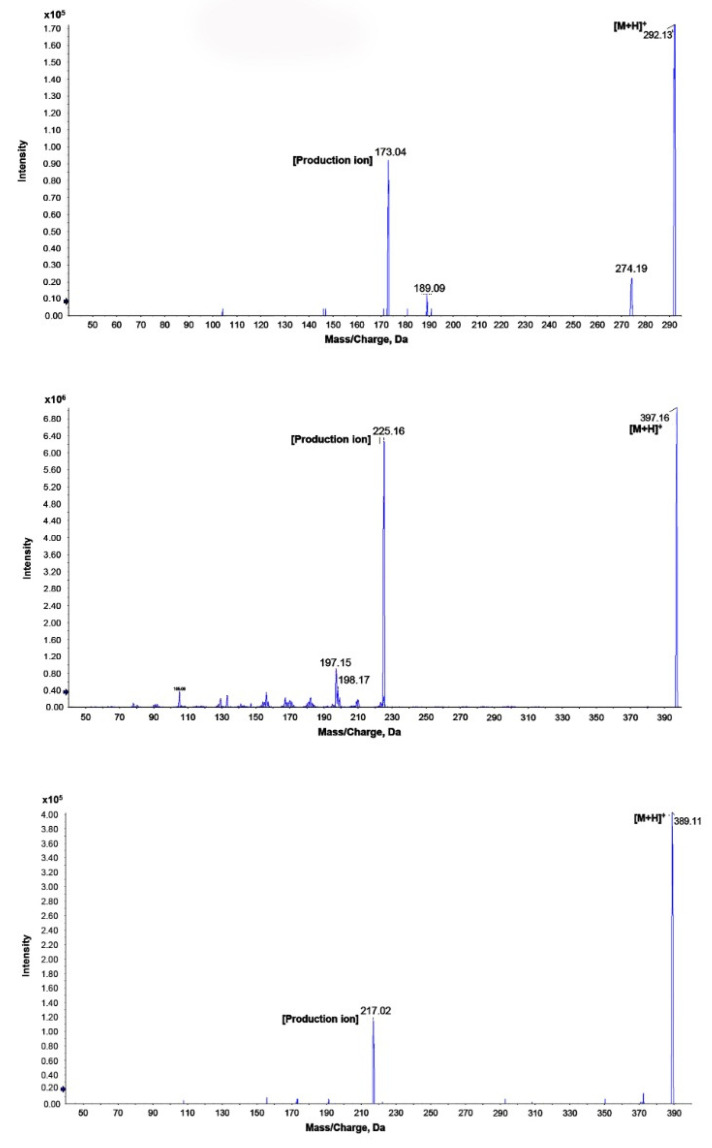
Representative mass spectra for AW-9a, WES-1, and WES-2 by the proposed liquid chromatography (LC)-MS/MS method.

Q1 full-scan mass spectrum was predominant at *m/z* 291.9 → 173.0, *m/z* 396.9 → 225.1, *m/z* 388.9 → 217.0, and *m/z* 146.9 → 91.0 for AW-9a, WES-1, and WES-2, respectively, as shown in Figure 4.

### 2.2. Method Validation

#### 2.2.1. Selectivity

Selectivity was assessed by comparing the chromatograms attained from six batches of blank human plasma samples with those spiked with low concentrations equivalent to the limit of quantitation (LOQ) of all studied drugs. No interference peak was detected at the retention times of 5.63, 1.05, 2.54, and 1.35 for AW-9a, WES-1, WES-2, and Coumarin (IS), respectively, indicating a high degree of method specificity.

#### 2.2.2. Calibration Curve and Quantitation Range

The linearity was assessed through spiking blank human plasma samples with standard solutions of all investigated chemicals along with the internal standard (IS). Peak area ratios of each drug to the IS were set as linear over a concentration range of 1–1000, 2.5–800, and 5–500 ng/mL for AW-9a, WES-1, and WES-2, respectively. The mean values (n = 3) for the regression equations obtained by the least square regression method (1/*x*^2^) by measuring the peak area ratio of the analytes to the IS were as follows: *y =* 0.011*x +* 0.018 (r^2^ = 0.999) for AW-9a, *y =* 0.0015*x +* 0.0014 (r^2^ = 0.999) for WES-1, and *y =* 0.006*x +* 0.053 (r^2^ = 0.992) for WES-2, as summarized in Table 1.

#### 2.2.3. Accuracy and Precision

The accuracy of the developed method, expressed as %Recovery, was found to range from 86.1 to 104.2% for all analytes. The average of intra-day variation in precision was less than 8.4%, over six replicates (n = 6). Similarly, the inter-day variations over eighteen replicates (n = 18) were less than 11.0%. The data obtained for inter- and intra-day variations of all the analyzed analytes from the quality control (QC) samples are presented in Table 2.

#### 2.2.4. Extraction Recovery and Matrix Effect

The relative recovery, also called the real recovery, was computed through matching the responses of peak area ratio for the spiked plasma samples before extraction to the post-extraction samples. The extraction recoveries of the three chemicals under investigation were also processed at the three QC levels (QCL, QCM, QCH) in six replicates, and the percentage recovery ranges from 89.45 ± 8.5% for AW-9a, 94.3 ± 4.9% for WES-1, and 86.66 ± 6.3% for WES-2. The matrix factor for all compounds at each QC concentration level ranges from 88.83 to 97.34%. These results revealed and confirmed that the protein precipitation method is suitable for quantifying analytes in human plasma as the IS-normalized matrix factor (CV%) was found to be less than 7.17%, which indicates suppression of the ions or that any enhancement from the plasma was nearly negligible, as shown in Table 3.

#### 2.2.5. Carry-Over

The carry-over effect was evaluated by injecting three blank plasma samples after the calibration curve at the QCH level of 800, 650, and 400 ng/mL for AW-9a, WES-1, and WES-2, respectively. No carry-over effects were observed as the peak area of the blank plasma samples after the analysis of the highest concentration of calibration standards at QCH was ≤20% of the first point on the calibration curve.

#### 2.2.6. Stability

The stability of the three drugs in human plasma was tested at low and high concentrations under various storage environments, with three determinations for each. Over various time periods, samples were subjected to varying temperature levels, and the results were compared with those obtained from the samples prepared freshly. It was found that plasma samples spiked with each drug were stable under subjected conditions of storage at room temperature for 24 h. The stability of the extracted plasma samples was also guaranteed in the auto-sampler conditions at 4 °C for 24 h. Freeze and thaw stability was assessed through three freeze-thaw cycles by storing samples at −80 °C, followed by thawing unassisted at room temperature. At the end of each cycle, samples were processed and analyzed, and means were calculated. Repeated freezing and thawing of plasma samples spiked with analytes at different QC (low and high concentrations of the three test compounds in plasma samples were used) showed negligible loss of the tested compounds during sample storage under different conditions at the analysis conditions, indicating a high degree of sample stability, as summarized in Table 4.

#### 2.2.7. Dilution Integrity

It was possible to cover the higher concentration limit and satisfactory precision and accuracy were achieved. The plasma samples were spiked at concentrations of 1600, 1300, and 800 ng/mL for AW-9a, WES-1, and WES-2, respectively; dilution folds were 1 to 2 and 1 to 4 at six determination per dilution. The accuracy results ranged from 95.17 to 99.66% for all compounds, while precision CV% was found to be between 1.90 and 8.75%. The integrity of all drugs up to fourfold dilution of concentrated plasma samples was revealed by the accepted values as summarized in Table 5.

## 3. Materials and Methods

### 3.1. Chemicals and Reagents

WES-1 and WES-2 were synthesized as reported previously [15]. Compound AW-9a was prepared through the reaction of (E)-3-(3-(dimethylamino)acryloyl)-2H-chromen-2-one [21] with aniline. The purity of the prepared compounds was greater than 96%, as determined by HPLC analysis. Coumarin (IS) was purchased from Sigma-Aldrich (St. Louis, MO, USA) with claimed purity of >99%. Formic acid (LC/MS grade), methanol, and acetonitrile (HPLC grade) were obtained from Fisher Scientific (Pittsburg, PA, USA). Ultra-pure water was obtained from MilliQ UF-Plus system (Millipore, Bedford, MA, USA); resistivity was maintained at 25 °C with TOC <5 ppb and >18 MΩ·cm^−1^. Blank human plasma samples were purchased from Sigma-Aldrich (St. Louis, MO, USA).

### 3.2. Instruments

Quantitative analyses of all investigated drugs in human plasma were carried out using an AB Sciex LC™ chromatographic coupled with a triple quadrupole API-3500 mass spectrometry system (Foster city, CA, USA) equipped with Turbo-ion spray operated in positive electrospray ionization (ESI) using MRM mode. The Analyst Hotfixes software version 1.6.3 (AB Sciex, Ontario, Canada) was acquired for data acquisitions to control all parameters of the HPLC and MS/MS and samples were quantified using the companion software MultiQuant 3.0 (AB Sciex, Ontario, Canada).

### 3.3. Mass Spectrometric Conditions

Air (zero grade) was the nebulizer gas, whereas nitrogen was used as the curtain, auxiliary, and collision gas. The source/gas-dependent parameters were as follows: curtain gas, 30 psi; collision gas, 8 psi; ion spray voltage, 4500 V; medium temperature, 500 °C; ion source gas one and gas two, 30 psi. The following precursor-to-product ion pairs used MRM transitions (*m/z*) performed in positive ionization mode at *m/z* 291.9 → 173.0, *m/z* 396.9 → 225.1, *m/z* 388.9 → 217.0, and *m/z* 146.9 → 91.0 for AW-9a, WES-1, WES-2, and Coumarin (IS), respectively, as represented in Table 6.

### 3.4. Preparation of Calibration Standards and Quality Control Samples

Primary stock solutions (1000 µg/mL) of AW-9a, WES-1, WES-2, and IS were prepared in DMSO/acetonitrile (1:10 *v/v*) and kept at −20 °C away from light. Working stock solutions were carried out by appropriate dilutions in acetonitrile to reach a concentration of 10,000, 8000, 5000, and 2500 ng/mL of AW-9a, WES-1, WES-2, and COM (IS), respectively. Another primary stock solution was diluted to prepare quality control (QC) samples for each analyte in blank plasma at three levels: low quality control (QCL), medium quality control (QCM), and high-quality control (QCH) samples at concentrations of 3, 50, and 800 ng/mL for AW-9a; 15, 200, and 650 ng/mL for WES-1; and 15, 120, and 400 ng/mL for WES-2, respectively.

### 3.5. Preparation of Fortified Samples

All plasma samples were stored frozen at −80 °C and were defrosted at room temperature prior to analyses. An aliquot quantity of 450 µL of human plasma either QC samples or calibration standards was placed into a scintillation vial, and fortified with 50 µL of working IS solution at 250 ng/mL and 50 µL of standard solutions of the chemicals under investigation to give required concentration. Precipitation of plasma protein was performed by adding 1.5 mL acetonitrile and tubes were agitated by vortexing and centrifuged at 5000 rpm for 10 min at 4 °C temperature. The upper clear supernatant layer from each scintillation vial was loaded into autosampler tray and 10 µL was injected into the chromatographic system for further analysis.

### 3.6. Assay Validation

As per U.S. Food and Drug Administration guidelines reported by Center for Drug Evaluation and Research (CDER), the developed bioanalytical method was fully validated with respect to linearity, selectivity, precision, accuracy, recovery, dilution integrity, matrix effect, and stability [22].

#### 3.6.1. Specificity

Blank human plasma samples were analyzed for the presence of any interfering peaks at the retention times of the compounds under investigation. Six separate batches of plasma samples were tested by comparing the chromatograms of drug-free plasma samples with others spiked with concentrations of the LOQ of AW-9a, WES-1, and WES-2 along with the IS to prove the lack of chromatographic interference from endogenous plasma components.

#### 3.6.2. Linearity

Linearity ranges were assessed by spiking 450 µL of human plasma samples with different concentrations over the range of 1–1000, 2.5–800, and 5–500 ng/mL for AW-9a, WES-1, and WES-2, respectively, along with 50 μL of 250 ng/mL COM (IS). Calibration plots were constructed using a weighted least-squares regression analysis (1/x^2^) by measuring the peak area ratio of the analytes to the IS. The blank samples and zero samples with IS were run with each calibration curve. The lower limit of detection (LLOD) was determined by successive analysis of spiked matrix with decreasing amounts of every standard until a signal-to-noise (S/N) ratio of 3:1 was reached, while the lower limit of quantitation (LLOQ) was defined as the lowest concentration of an analyte that can be quantified with acceptable precision and accuracy. Moreover, the set of acceptance criteria deviated ±15% from the nominal value for back-calculated standard concentrations, except for LLOQ, which was set as ±20%.

#### 3.6.3. Accuracy and Precision

Intraday accuracy and precision were determined via analysis of the fortified samples at three levels of concentration (QCL, QCM, and QCH) within the same day at six replicates (n = 6). Meanwhile, the inter-day precision was evaluated through analyses of the samples at three concentration levels of QC samples via repeating analysis over three days (n = 18). The actual concentration at each standard solution was computed from the ratios of the area under the peak to that of IS and then matched with the nominal values. Precision was evaluated by relative standard deviation (%RSD) values, which should be less than 15%, while accuracy was expressed as the coefficient of variation with an acceptance criterion ±15% from the nominal values.

#### 3.6.4. Matrix Effect and Extraction Recovery

The examination of matrix effect was assessed using the matrix factor by calculating the ratio of the peak area in the presence of matrix (spiked sample with analytes) to the peak area in neat pure solution of analytes in six replicates at the three different QC concentration levels: 3, 50, and 800 ng/mL for AW-9a; 15, 200, and 650 ng/mL for WES-1; and 15, 120, and 400 ng/mL for WES-2. Similarly, IS-normalized matrix factor estimation was obtained by dividing the matrix factor of each analyte over the IS matrix factor.

#### 3.6.5. Dilution Integrity

The dilution effect was investigated to ensure that plasma samples could be efficiently diluted with blank plasma without affecting the concentration of the investigated drugs. Plasma samples were spiked with high concentration of 1600, 300, and 800 ng/mL for AW-9a, WES-1, and WES-2, respectively; dilution folds were 1 to 2 and 1 to 4. Three replicates of each dilution were examined and replicates had accuracy of 100 ± 15% and precisions of ≤15%.

#### 3.6.6. Stability Studies

QC samples spiked at upper and lower concentration levels were analyzed at six replicates in order to evaluate the stability of each drug in human plasma. QC samples were exposed to different conditions; the first one was performed in the injection medium (samples after extraction were kept in the auto-sampler at 4 °C for 24 h before injection into the system), and the second is stability of the three analytes in plasma after exposure at room temperature for 3 and 6 h (bench top stability). It was examined at QCL and QCH levels in triplicates. Finally, freeze and thaw stability following three freeze–thaw cycles at −80 °C and stability at 4 °C for 3 consecutive days (24 h, 48 h, 72 h) of the analytes in the plasma were determined using QC samples spiked with AW-9a, WES-1, and WES-2. Recommended mean concentrations at each level are ±15% of the nominal level.

## 4. Conclusions

This article describes the development and validation of a new analytical LC-MS/MS method for the quantification of three new carbonic anhydrase inhibitors in human plasma. The validation data indicate that the developed method is highly sensitive, accurate, simple, and precise. Once in vitro toxicology studies are complete, the developed method can be easily utilized to study the pharmacokinetics of these new potential anti-cancer agents in animal models as part of pre-clinical studies.

## Figures and Tables

**Table 1:** Linearity results for AW-9a, WES-1, and WES-2 in human plasma. LLOQ, lower limit of quantitation; LLOD, lower limit of detection.

	Intercept		Slope		R^2^		LLOQ	LLOD	Linearity Range
	Average *	SD	Average *	SD	Average *	SD	(ng/mL)		(ng/mL)
AW-9a	0.018	0.0116	0.011	0.006	0.9991	0.0002	1	0.03	1–1000
WES-1	0.0014	0.0004	0.0015	0.001	0.9991	0.0005	2.5	1	2.5–800
WES-2	0.053	0.0270	0.006	0.0003	0.992	0.006	5	1.5	5–500

* Average of three determination.

**Table 2:** Intra- and inter-day accuracy and precision results for AW-9a, WES-1, and WES-2 *.

Analyte	Concentration		Intra-Day		Inter-Day	
	(ng/mL)		Recovery (%)	CV (%)	Recovery (%)	CV (%)
AW-9a	LLOQ	1	103.5	5.35	99.20	4.55
	QCL	3	93.3	6.40	94.44	5.17
	QCM	50	99.3	5.69	98.97	5.19
	QCH	800	100.4	8.44	99.71	7.61
WES-1	LLOQ	2.5	86.05	7.86	90.15	4.91
	QCL	15	104.2	2.01	102.3	5.01
	QCM	200	98.40	1.24	99.72	2.31
	QCH	650	94.82	8.30	91.24	10.8
WES-2	LLOQ	5	94.32	4.35	93.87	4.1
	QCL	15	99.38	2.66	97.90	5.18
	QCM	120	97.34	1.77	98.38	1.63
	QCH	400	96.81	1.15	97.04	2.91
N			6		18	

* Accuracy is expressed as the Recovery% with an acceptance criterion ±15% from the nominal values, while precision is expressed as a percentage of coefficient of variation (CV (%)), required to be not more than 15%. LLOQ; lowest limit of quantitation, QCL; quality control low, QCM; quality control medium, QCH; quality control high.

**Table 3:** Matrix effects for AW-9a, WES-1, and WES-2 in human plasma.

Analyte	Concentration		Extraction Recover (%)		Matrix Effect	
	(ng/mL)		Mean	RSD (%) *	CV (%)	RSD (%)
AW-9a	QCL	3	82.31	12.2	7.65	5.13
	QCM	50	89.91	4.20	7.17	7.11
	QCH	800	96.13	9.13	8.20	7.05
WES-1	QCL	15	98.74	5.60	6.31	6.10
	QCM	200	96.70	3.50	5.22	6.15
	QCH	650	87.59	5.74	5.39	6.32
WES-2	QCL	15	92.77	6.29	2.66	6.15
	QCM	120	83.61	8.00	0.71	5.57
	QCH	400	83.60	4.64	2.91	6.86

* RSD; relative standard deviation, QCL; quality control law, QCM; quality control medium, QCH; quality control high.

**Table 4:** Stability results for AW-9a, WES-1, and WES-2 in human plasma at different conditions.

Analyte	Concentration (ng/mL)		Short Term Stability at Room Temperature (24 h)			Freeze and Thaw Stability at −80 °C (3 cycles)			Processed Sample Stability at 4 °C (72 h)		
			Accuracy (%)	CV (%)	Stability (%)	Accuracy (%)	CV (%)	Stability (%)	Accuracy (%)	CV (%)	Stability (%)
AW-9a	QCL	3	96.76	4.67	89.28	90.92	2.89	86.28	102.79	13.99	98.85
	QCH	800	100.81	7.06	94.15	105.94	6.71	101.32	100.20	4.73	95.81
WES-1	QCL	15	86.02	1.05	97.37	89.30	3.97	96.86	93.38	6.91	95.77
	QCH	650	88.69	0.86	94.51	91.93	5.11	111.37	90.00	3.35	95.77
WES-2	QCL	15	89.63	4.10	91.22	87.92	4.09	91.95	84.58	3.87	97.29
	QCH	400	94.12	1.32	99.96	95.33	2.08	98.69	92.27	6.48	101.13
N			3			3			3		

**Table 5:** Evaluation of the dilution integrity of AW-9a, WES-1, and WES-2 in human plasma.

Analyte	Spiked Concentration (ng/mL)	Dilution Fold	Accuracy (%)	CV (%)
AW-9a	800	1:2	98.16	8.75
		1:4	95.17	7.36
WES 1	650	1:2	89.54	2.50
		1:4	89.67	1.90
WES 2	400	1:2	95.65	2.83
		1:4	99.66	6.44

**Table 6:** LC-MS/MS parameters selected for the quantification of AW-9a, WES-1, WES-2, and Coumarin (IS).

Analyte	Q1 ^a^ (*m/z*)	Q3 ^b^ (*m/z*)	DP ^c^ (V)	EP ^d^ (V)	CE ^e^ (V)	CXP ^f^ (V)
AW-9a	291.978	173.037	81.0	10	40	16
WES-1	396.979	225.140	80	10	90	8
WES 2	388.930	217.056	111	10	35	8.0
IS	146.941	91.030	70.0	10	35	12

^a^ Q1, precursor ion; ^b^ Q3, product ion; ^c^ DP, declustering potential; ^d^ EP, entrance potential; ^e^ CE, collision energy; ^f^ CXP, cell exit potential.
